# Development of high-speed precision maize metering device for dense planting pattern with standard ridges

**DOI:** 10.3389/fpls.2024.1452699

**Published:** 2024-11-11

**Authors:** Junzhi Chen, Tianyuan Guan, Zixin Yuan, Fudong Xu, Kuangyu Zhao, Han Tang, Jiale Zhao, Jinwu Wang

**Affiliations:** ^1^ College of Engineering, Northeast Agricultural University, Harbin, China; ^2^ Quality Inspection Department, Fang Zheng Comprehensive Product Quality Inspection and Testing Center, Harbin, China; ^3^ College of Biological and Agricultural Engineering, Jilin University, Changchun, China

**Keywords:** dense planting pattern with large ridges, double row seeding, maize metering device, response surface, energy consumption

## Abstract

This study proposed a high-speed precision dual-chamber maize metering device for the dense planting pattern with standard ridge promoted in China. Through theoretical analysis of the sowing process, parameters for the key components have been designed. The metering device is capable of planting two rows in a single pass for high-speed precision seeding. The effect of operating speed and negative pressure on seed metering quality was investigated. A high-speed camera was used to capture the trajectory of maize seed at different operating speeds, and it was found that intra-row shifts were caused by collisions at the mouth of the seed guide tube and rotation of the seed as it fell. Employing a two-factor, five-level orthogonal rotation test, Investigated the optimal operating parameters for the maize metering device. Response surface analysis showed that optimum seed metering quality was achieved at 14.2 km/h and 13.5 kPa. Validation tests showed a qualified rate of 97.93%, with a coefficient variation of 8.97% for this method. Additionally, an energy consumption analysis indicated a reduction in operating energy consumption of approximately 32% compared to conventional air suction seed metering devices for dense planting with large ridges on the same area of farmland. This study provides insights for reducing energy consumption in the seeding process, contributing to the sustainable development of agricultural resources.

## 1 Introduction

### 1.1 Introduction of seed metering device

As global arable land continues to decrease in size and the world population steadily increases, immense pressure arises due to limited resources, projected to reach saturation by 2050 (FAO (Food and Agriculture Organization), 2018). China, a superpower boasting a population exceeding 1.4 billion, has less than half the global average per capita arable land and less than one-third of the world’s per capita water supply (Ministry of Natural Resources of the People’s Republic of China, 2021). Enhancing resource utilization, adopting cleaner agricultural production methods, and producing more food on limited land pose significant challenges.

Among the three major cereal crops, maize has the highest total yield, and increasing its production is a key means to ensure food security (Lu and Lu, 2017; Yuan et al., 2018; Song et al., 2022; Li and Tao, 2023). It is worth emphasizing that in northeastern China, a method known as the dense planting pattern with large ridges has been widely promoted and effectively applied to achieve increased yields through the adjustment of planting density using dislocation sowing technology (Ministry of Agriculture and Rural Affairs of the People’s Republic of China, 2022). In comparison to traditional maize ridge planting patterns, this technique involves dense planting with intercropped planting, increasing inter-row spacing, and providing water and fertilizer in stages and directionally. This approach improves ventilation and light conditions between plants, significantly enhances water and fertilizer use efficiency, and increases land resource utilization efficiency. It is essential for the sustainable development of agriculture.

Precision planting is crucial in the grain production process as a major contributor to stable and increased yields (Han et al., 2018; Cisternas et al., 2020; Wang et al., 2023; Tang et al., 2024). High-speed precision metering devices are the core tools for advancing precision seeding technology, directly influencing the seeding quality (Lu et al., 2016; Tang et al., 2022; Wen et al., 2023; Xu et al., 2023). Seed metering devices can be classified into mechanical and pneumatic types based on their working principles (Jia et al., 2018; Li et al., 2022; Wang et al., 2022; Tang et al., 2023b). Mechanical metering devices do not to achieve high operating speeds during operation, and being prone to seed damage (Wang et al., 2017; Li et al., 2023c, 2023b). In contrast, pneumatic metering devices use air currents to capture seeds, which can effectively reduce damage to the seeds and operate at faster speeds with higher precision (Gao et al., 2021; Karayel et al., 2022; Li et al., 2023a; Yatskul and Lemiere, 2018).

In order to improve the operational quality of seed metering devices, a wide range of studies from different perspectives have been carried out by scholars all over the world. Zhao et al. (2024) proposed a centrifugal filling-cleaning seed metering device. The study analyzed the effect of major factors including the turbine structure, air pressure and operating speed on the sowing performance Under operating conditions at a speed of 18 km/h, the qualified rate exceeded 90%, the qualified rate refers to the proportion of seeds that are successfully sown and correctly positioned during the sowing process. Li et al. (2023) designed a maize seeder using centrifugal slurry cleaning. The seeding tray and front shell are designed so that the seed can use the centrifugal force for filling, cleaning, releasing and returning. When the speed is in the range of 10-20 km/h, the qualified rate can be greater than 90%. Du and Liu (2023) developed a metering device utilizing air pressure and gravity-assisted filling. They optimized the parameters of the metering disk through simulation experiments, achieving a qualified rate exceeding 90% at operational speeds of 8 - 16 km/h. Tang et al. (2023a) designed an aerodynamic maize metering device with an inclined metering disk. The study examined the effect of seed metering system operating parameters on metering quality, achieving a qualified rate of 98.31% and a coefficient variation of 9.26% at an operating velocity of 10 km/h.

However, there is a scarcity of research on seed metering devices specifically designed for special planting patterns, such as the maize-soybean intercropping pattern and the dense planting pattern with standard ridges. In recent years, the dense planting pattern with large ridges for maize has started to gain widespread application. The seed metering devices used in this context are often custom adaptations of existing seeding machines, where specific components or settings have been altered to accommodate the unique requirements of special planting patterns. Unfortunately, these adaptations fall short of ensuring a uniform plant spacing distribution, a necessity in agronomy, leading to unnecessary wastage of critical agricultural resources such as seeds, water, fertilizers, and pesticides.

### 1.2 Introduction of planting pattern

**Figure 1:**
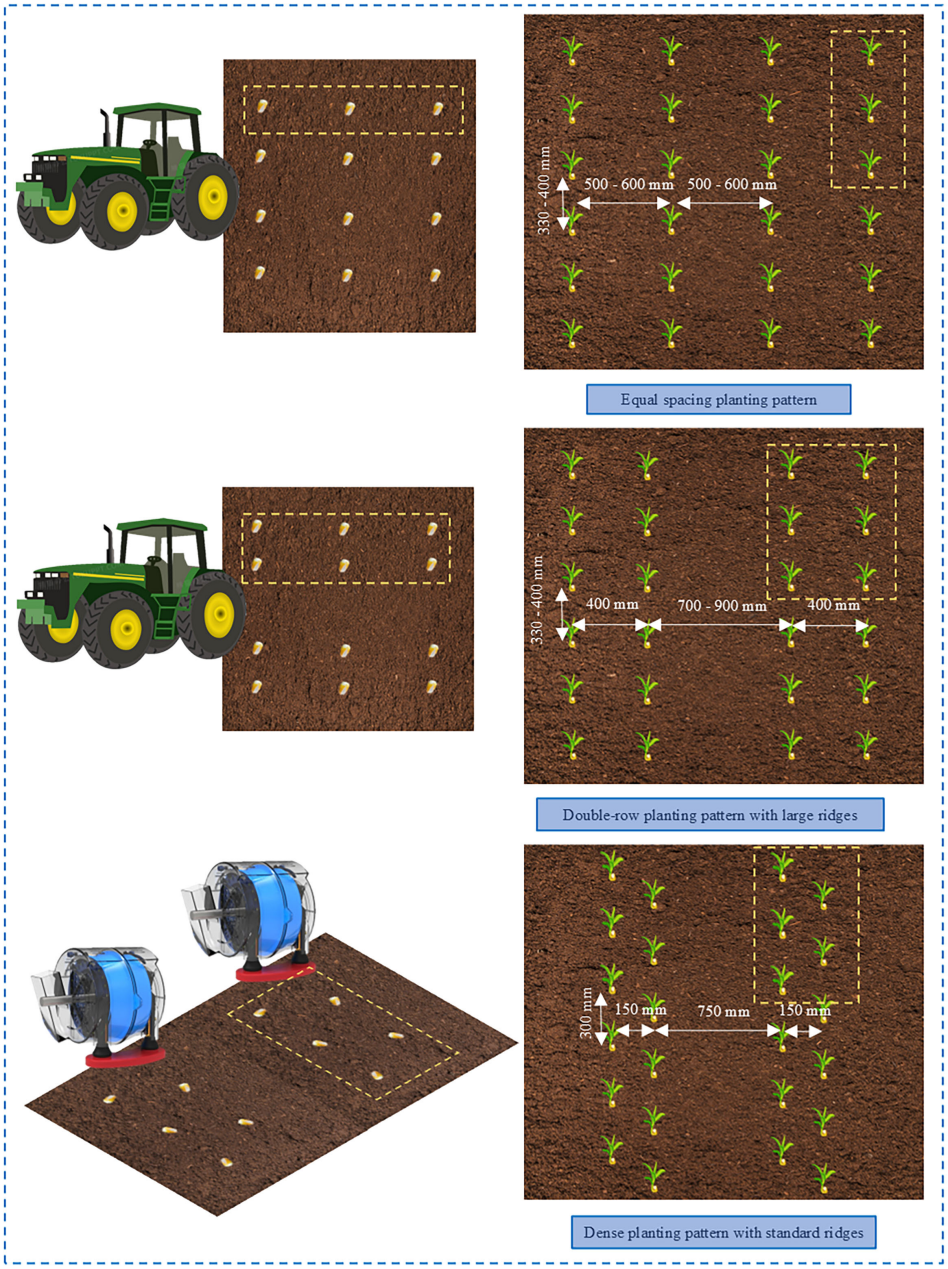
Maize planting pattern.

Maize is predominantly grown in China using two ridge planting patterns: equal spacing planting and large ridges double-row planting. For equal spacing planting, the agricultural requirements are a row spacing of 500 - 600 mm and a plant spacing of 330 - 400 mm. The double-row planting pattern with large ridges involves merging two traditional ridges into one and planting double rows with a small inter-row spacing of 400 mm and a large inter-row spacing of 700 - 900 mm. However, this pattern has some agronomic and land utilization shortcomings. The dense planting pattern with standard ridges are planted in a triangular staggered manner, with a reduced inter-row spacing of 150 mm and a front-to-back plant spacing of 300 mm. The large inter-row spacing is adjusted to 750 mm (**Figure 1**). This planting pattern, known as the dense planting pattern with large ridges, has been promoted and applied widely in the northeast region of China, achieving good results The root area of the crop was significantly increased by rational, dense, staggered sowing. This improves air circulation and improves the transmission of light between plants.

The dense planting pattern with large ridges holds significant importance for clean maize production. Firstly, this pattern maximizes the optimization of land space layout, achieving higher planting density and consequently obtaining greater yields on limited land. By fully utilizing land resources, it enhances production efficiency, reduces excessive land development, and minimizes the ecological impact on the soil environment. Secondly, the dense planting pattern with large ridges can reduce the usage of pesticides and fertilizers. The staggered arrangement of plants weakens competition between crops and reduces the risk of pest and disease transmission, thereby decreasing reliance on chemical pesticides. Additionally, with higher planting density, fertilization requirements become more precise, avoiding over-fertilization and reducing agricultural pollution. Furthermore, the dense planting pattern with standard ridges helps improve water use efficiency. Through a rational staggered layout, soil moisture evaporation is reduced, soil water retention capacity is enhanced, and irrigation demands are lowered. This aids in more effectively managing water resources in regions with limited water, providing significant advantages in water resource conservation.

The dense planting pattern with standard ridges demonstrates significant advantages in environmental protection and resource conservation. By optimizing land utilization, reducing pesticide and fertilizer usage, and improving water use efficiency, this pattern provides a more intelligent and eco-friendly choice for agricultural clean production and sustainable development.

In this study, a high-speed precision dual-chamber maize metering device is proposed for the dense planting pattern with standard ridges. In this study, the seeding process was analyzed to determine the critical structural parameters of the metering device. Employing high-speed camera technology enabled the comparison of seed sowing trajectories at various operating speeds. Response surface methodology was utilized to optimize the parameters, enhancing operational performance. This study aims to offer a reference for the design of seed metering devices tailored to specific planting patterns, and to bolster the sustainability of agricultural production systems.

## 2 Materials and methods

### 2.1 Structure and working principle

**Figure 2:**
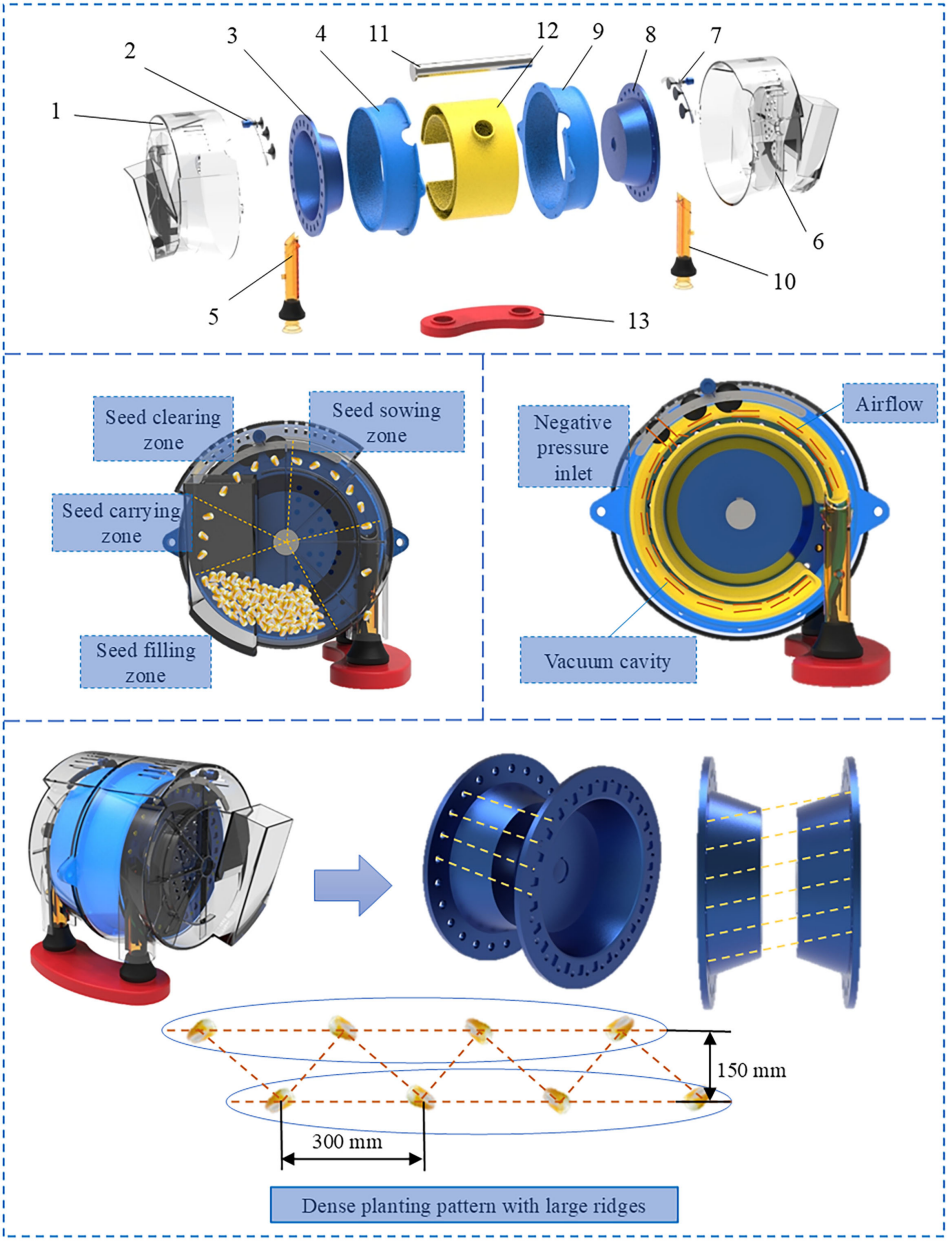
Structure and working principle. 1. Outer casing I, 2. Seed clearing wheel I, 3. Seed disc I, 4. Inner casing I, 5. Seed guide tube I, 6. Outer casing II, 7. Seed clearing wheel II, 8. Seed disc II, 9. Inner casing II, 10. Seed guide tube II, 11. Transmission shaft, 12. Suction ducts, 13. Seed guide tube bracket.

The structure of the high-speed precision dual-chamber maize metering device comprises seed guide tube, transmission shaft, suction ducts, inner casing, seed discs, seed clearing wheels, and outer casing (**Figure 2**). The dual seed discs are installed in a staggered manner on the transmission shaft. The suction ducts are fixed to the inner casing, aligning with the two seed discs to create an air chamber. The seed-clearing wheels are precisely positioned and mounted in designated holes on the outer casing, ensuring proper alignment with the seed discs and effective seed clearance. The seed guide tube is installed at the sowing zone edge.

During operation, the seed is stored in a seed filling area located inside the outer housing. A drive shaft rotates the alternately mounted twin seed discs. The fan creates a pressure differential between the outside of the seed discs and the air chamber, causing the seed to attach to the holes and move with the rotation of the seed discs. When the seeds reach the seed clearing area, they are bumped and squeezed by the seed clearing wheel. The individual seeds with the strongest adhesion are retained, while the excess seeds fall off and return to the seed filling zone. These individual seeds continue to rotate with the holes until they reach the end of the suction duct. At this point, the adhesion force diminishes, and the seeds fall from the holes through the seed guide tube onto the seedbed, completing the sowing operation. Using dual plates to sow on a single ridge enables higher operational speeds at lower rotation speeds, reducing energy consumption and promoting cleaner maize planting practices.

### 2.2 Analysis of filling process

During the filling process, seeds are more effectively filled into the holes when the holes remain in the filling zone for a longer period, enhancing the overall filling efficiency. Under conditions of constant operating speed, the filling time is related to the quantity of holes and the range of the seed filling zone. To optimize the seed filling process, the angle occupied by the seed filling zone was expanded by adjusting the size of the seed disc and the position of the seed guide tube relative to the seed disc.

The size of the maize metering device is primarily determined by the diameter of the seed discs, which in turn influences parameters such as seed velocity and centrifugal force, depending on the distance from the hole to the center of the disc. Therefore, the diameter of the seed discs should not be too large. Otherwise, the seed velocity and centrifugal force in the metering holes will be too high, making it difficult to ensure stable seed adsorption under high-speed conditions. If the seed disc diameter is too little, the maximum quantity of seeds carried by the discs will be reduced. At the same operating speed, a faster rotation speed of the discs will be required, consuming more energy. Currently, the design range of the seed discs diameter is 140 - 260 mm, with the diameter of 156 mm determined for the location of the holes. The seed disc diameter should be sufficient to accommodate the seed clearing mechanism and have sufficient outer edges. It has been determined that the seed disc diameter should be 180 mm.

Under conditions where the seed spacing and operating speed are constant, increasing the quantity of holes can reduce the rotation speed of the plates and decrease energy consumption. This facilitates better seed adsorption. However, it is not necessarily better to have more holes, and the optimal quantity of holes for most pneumatic maize metering devices is around 26. To meet the demands of high-speed precision seeding in maize, while respecting the size constraints of this metering device, it was finally decided to use 25 holes. For stable adsorption of seeds, the following conditions must be met:


(1) $$P\frac{d}{2}\geq QT$$

Where, *Q* is the combined force of centrifugal and frictional resistance, *d* is diameter of holes, *P* is the adsorption force, and *T* is distance from seed center of mass to disc.

**Figure 3:**
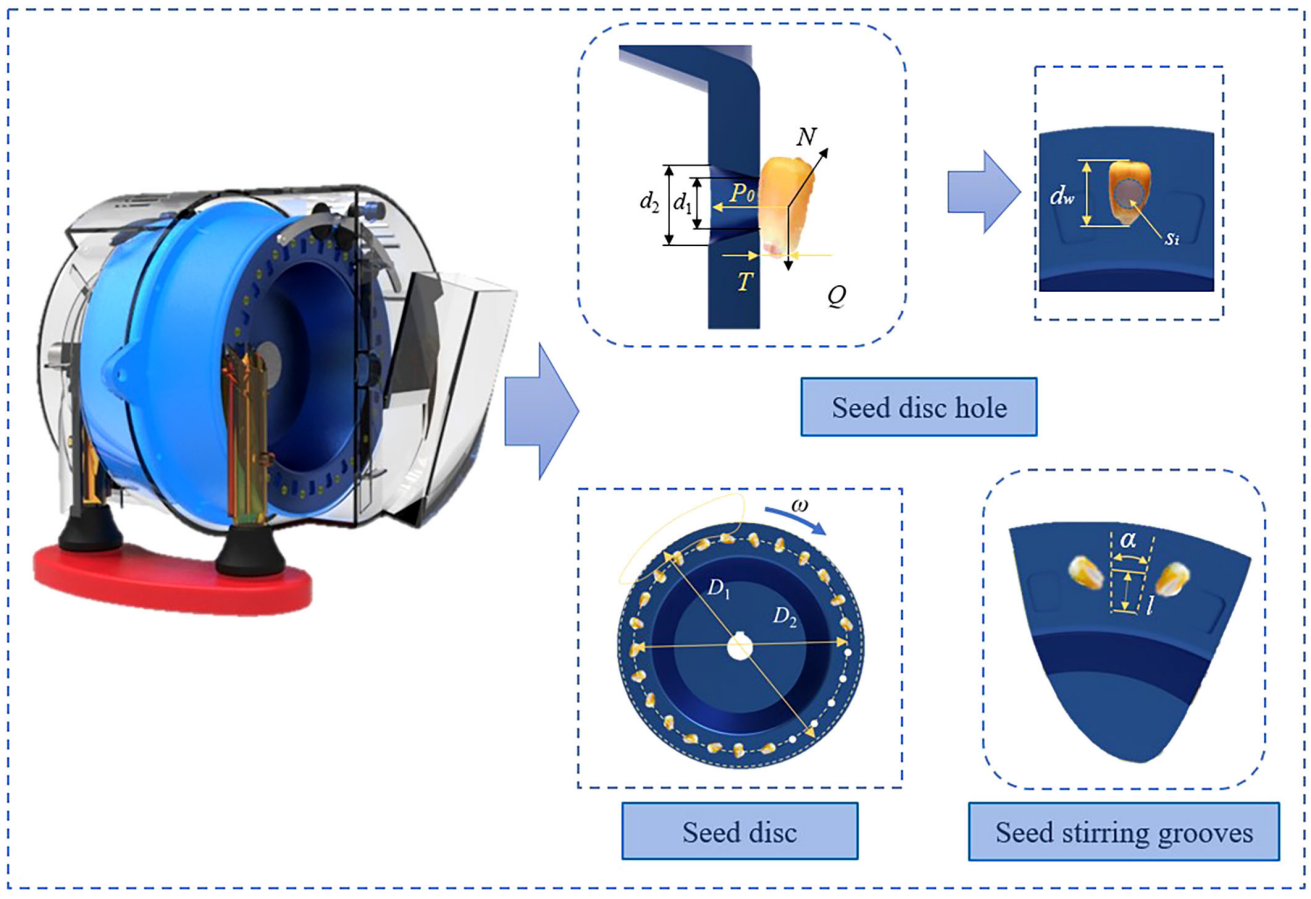
Analysis of filling process.

In addition, referring to the typical diameters of holes in pneumatic maize seed metering devices, which are normally between 4 - 5.5 mm, the diameters of the holes were set at *d*_1_ = 5 mm and *d*_2_ = 7.5 mm (**Figure 3**).

Seeds at different positions in the seed filling zone experience different forces. Seeds at the bottom of the population and near the holes are subjected to greater force from the flow field. Conversely, seeds located at the outermost fringe of the seed population, closer to the seed clearing zone, experience minimal force from other seeds and are more easily adsorbed. The chances of seed adhesion gradually decrease as the seed moves towards the inside. Reducing the interaction forces within the seed population and enhancing the activity of seeds within the population can help improve the filling probability. Employing disturbance mechanisms uniformly distributed around the periphery of the seed discs to enhance the filling effect. The transverse stirring grooves correspond one-to-one with the holes, and their structural parameters are designed based on the maize average size, the angle of single seed stirring tank were set at *α* = 13° and the length of single seed stirring tank were set at *l* = 9 mm.

### 2.3 Analysis of clearing process

The clearing mechanism is a key tool in reducing the multiples rate, enabling precise seeding by separating individual seeds. The clearing process should commence after stable seed adhesion, and the installation position of the seed cleaning wheels allows the excess cleaned seed to drop into the seed filling zone while preventing excessive cleaning.

Assuming the seed clearing process starts from point *M*. The seed clearing process should ensure that when the seed from the previous hole falls, it does not collide with the seed adhered to the next hole. Therefore, ensure that the horizontal distance between seeds is greater than the maximum maize seed size. According to previous measurements of maize seed sizes, the maximum size of a single maize seed is 13.15 mm, rounded up to 14 mm. Therefore, the mounting position of the seed clearing wheel should satisfy the requirements:


(2) $$L_{1}-L_{2}=r\cos\varphi_{1}-r\cos(\varphi_{1}+\frac{2\pi }{n})>14mm$$

Where *φ*_1_ is the starting position angle for the seed clearing wheels installation, *L*_1_ is the horizontal distance from the center of the seed disc to the center of the first hole at the start of seed clearing, *L*_2_ is the horizontal distance from the center of the seed disc to the center of the next hole, *n* is the quantity of holes. It is determined that *φ*_1_ > 36.74°, so *φ*_1_ was set to 40°.

The seed clearing wheels should gradually approach the holes. As the seed clearing process progresses, the clearing intensity should gradually increase to remove excess seeds from the holes. The clearing process is conducted intermittently in three stages. The parameters of the seed clearing wheels are mainly determined by the horizontal distance and angle between the center of the holes and seed clearing wheels. To achieve effective seed clearing, it is essential to ensure that the seeds return to their adhesive state after passing through each stage of clearing. Hence, the distance between every two seed clearing wheels must be longer than the maximum length of a maize seed, and Δ*θ* should satisfy:


(3) $$\text{Δ}\theta >\arccos\frac{l_{2}^{OA_{1}}+l_{2}^{OB_{1}}-14^{2}}{2l_{OA_{1}}l_{OB_{1}}}$$

To ensure the stability of seed clearing under the condition of gradually increasing clearing intensity at each level, the duration of clearing at each level is increased equally, that is, the angle occupied by each clearing wheel gradually increases. To make optimal use of the clearing area, based on theoretical analysis and preliminary test results, the final diameters of the three levels of clearing wheels were determined to be 19, 21, and 23 mm, with Δ*θ* set at 15°.

**Figure 4:**
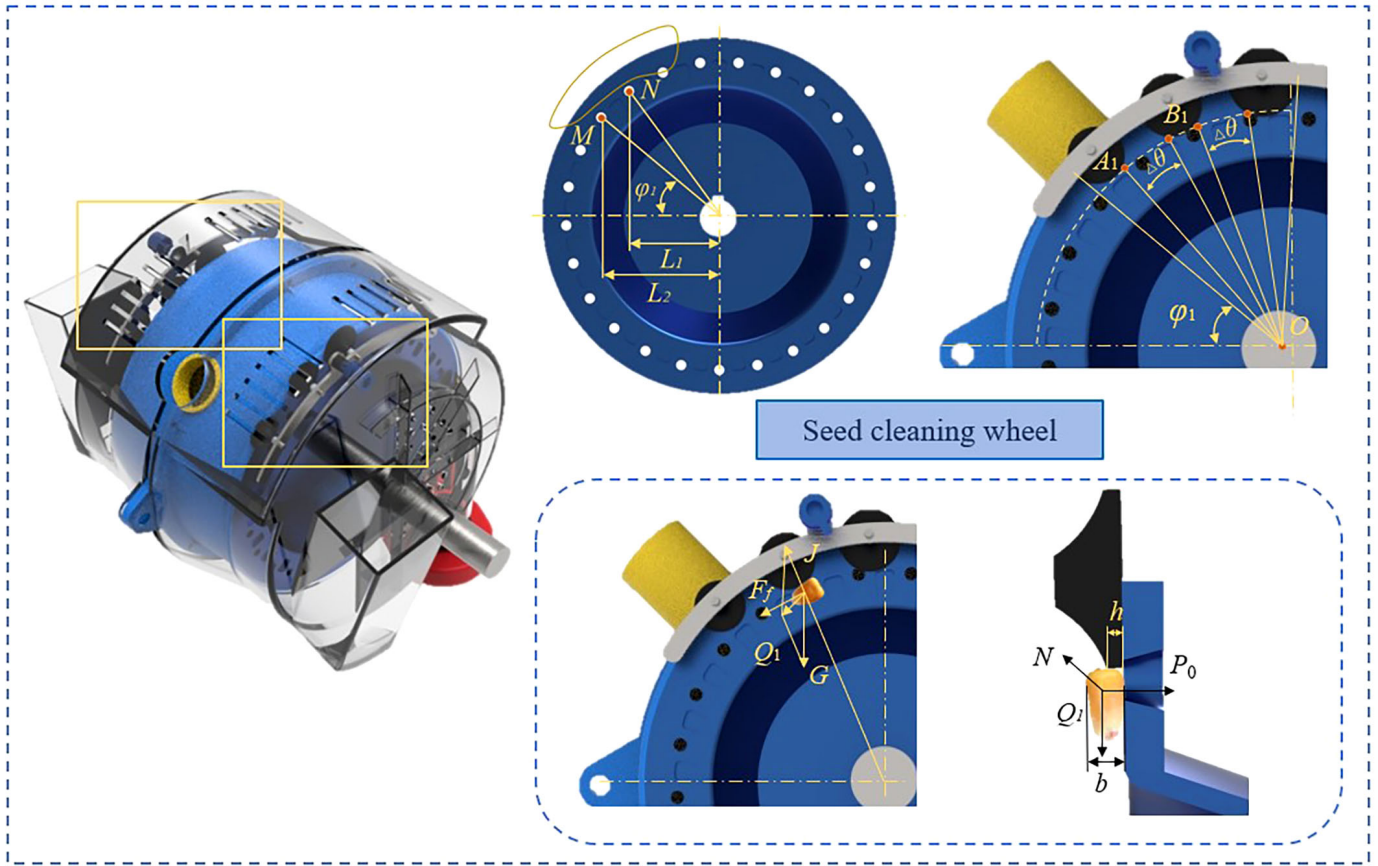
Analysis of clearing process.


The seed clearing process involves ensuring the stable movement of individual seeds with adsorption advantages. In high-speed operation of the metering device, seeds move rapidly. It is crucial to effectively clear seeds without excessive removal to ensure seed metering quality. The force exerted on the seed at the moment it clears and comes into contact with the edge of the seed clearing wheel (**Figure 4**). To ensure that seeds with the best retentive advantage are not cleared away, it is necessary to satisfy the following conditions:

(4) $$\frac{\pi d^{3}}{8}P_{0}\geq Q_{1}(\frac{b}{2}-h)$$

Where, *P*_0_ is pressure difference on either side of the hole, *Q*_1_ is resultant force of centrifugal force, gravity, and frictional resistance acting on the seed, *d* is size of holes, *b* is thickness of the maize, *h* is thickness of the seed clearing wheel.

### 2.4 Analysis of sowing process

**Figure 5:**
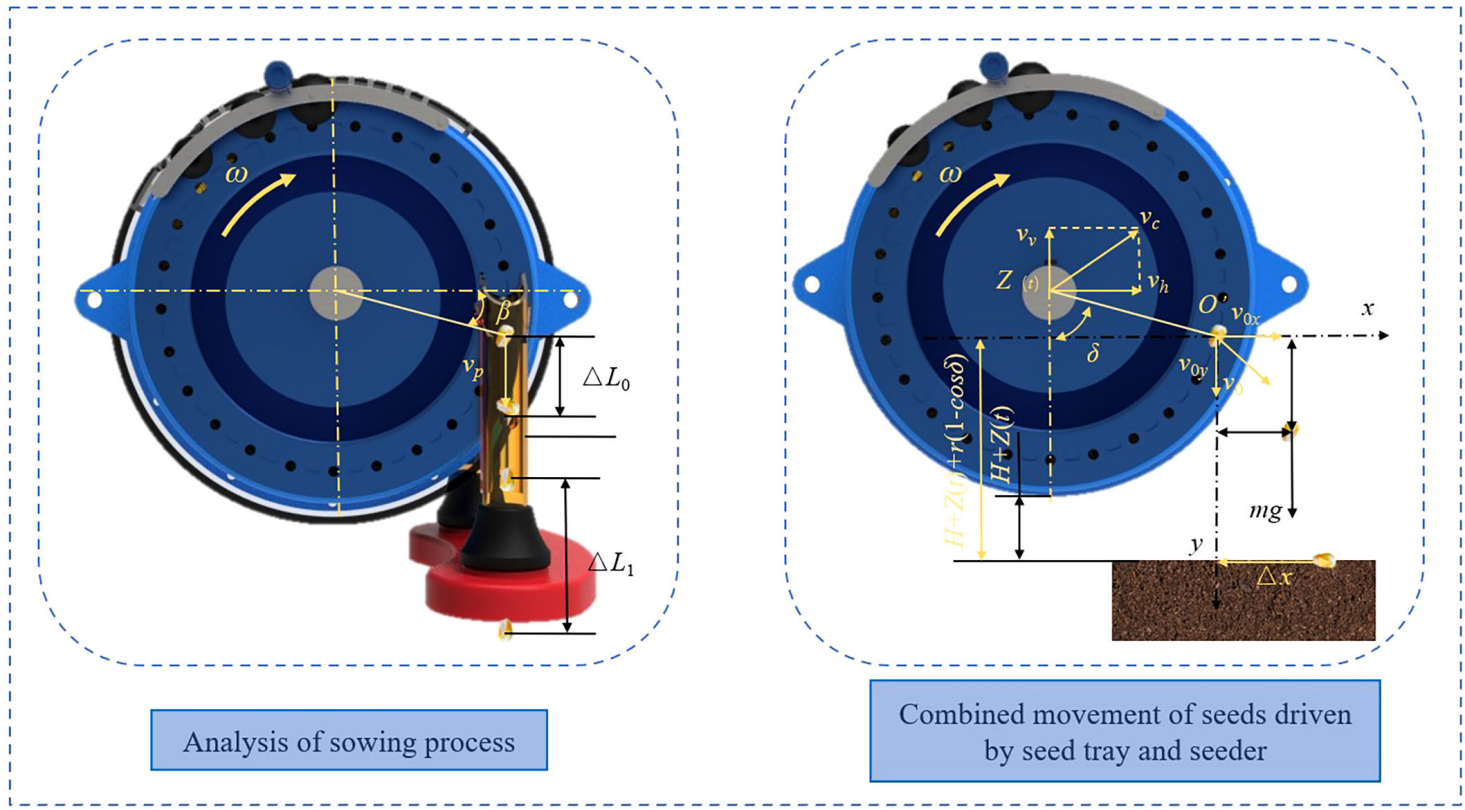
Analysis of sowing process.

Operating speed has a significant effect on sowing uniformity. As operating speed increases, plant spacing becomes less uniform, which can cause multiple or missed seeding, resulting in a lower qualified rate. To examine the impact of operating speed on the process of sowing, a kinematic analysis was conducted on the process of seed sowing (**Figure 5**).

As the speed of operation rises, the spacing between adjacent seeds decreases, leading to an increase in instances of multiple seedings or misses due to inconsistent seeding positions, thereby affecting the seed metering quality. As the spacing between adjacent seeds decreases during the seed descent process, seeds deviating from the trajectory are more prone to collision and displacement within the seed delivery tube, resulting in multiples or misses, deteriorating the uniformity of plant spacing and impacting the seeding performance.

Once the seeds are released from the seed discs and enter the seed guide tube, they accelerate and move downward along the vertical direction:


(5) $$\text{Δ}L=\frac{2\pi r}{Z}+\frac{162aS^{2}}{25v^{2}}+\frac{18ax}{5v}t$$

Where Δ*L* is the spacing between adjacent seeds during the descent process, *Z* is the number of holes, *r* is the radius at the location of the hole on the seed disc, *a* is the acceleration of the seeds during descent, *x* is the theoretical spacing, *v* is the planter operating speed.

An analysis of the sowing process suggests that to reduce excess bouncing and uneven sowing during seeding, it is beneficial to impart a horizontal component to the seed descent velocity, counteracting the operating speed of the planter to achieve zero-velocity seeding.

During the sowing process, the seeds undergo a combined motion driven by both the seed disc and the planter (**Figure 5**). As the planter advances, vibrations originating from the unevenness of the ground are directly transmitted to the planter, with such vibrations becoming more pronounced as the velocity increases. Alterations in the velocity and displacement in the y-direction affect the seeding spacing and interval time, and as the magnitude of the vibrations increases, this influence is further exacerbated.


(6) $$\text{Δ}x=\frac{v_{0x}}{g}\left[-v_{0y}+\sqrt{v_{0y}^{2}+2gH_{h}+2gZ(t)+2gr(1-\cos\delta )}\right]$$

Where, *H_h_* is the distance between the bottom of the seed disc and the ground, *Z*(*t*) is the displacement of the seed disc center caused by vibration, *δ* is the angle between the sowing direction and the vertical direction.

The analysis above indicates that the horizontal distance of seed sowing is related to rotational speed, the radius of the seed disc, vibration magnitude and frequency, sowing height, and angle. To ensure a smooth and orderly seed sowing process, it is necessary to design the sowing position in a reasonable manner. This will improve the quality of the sowing process.

### 2.5 Bench test

#### 2.5.1 Test environment

**Figure 6:**
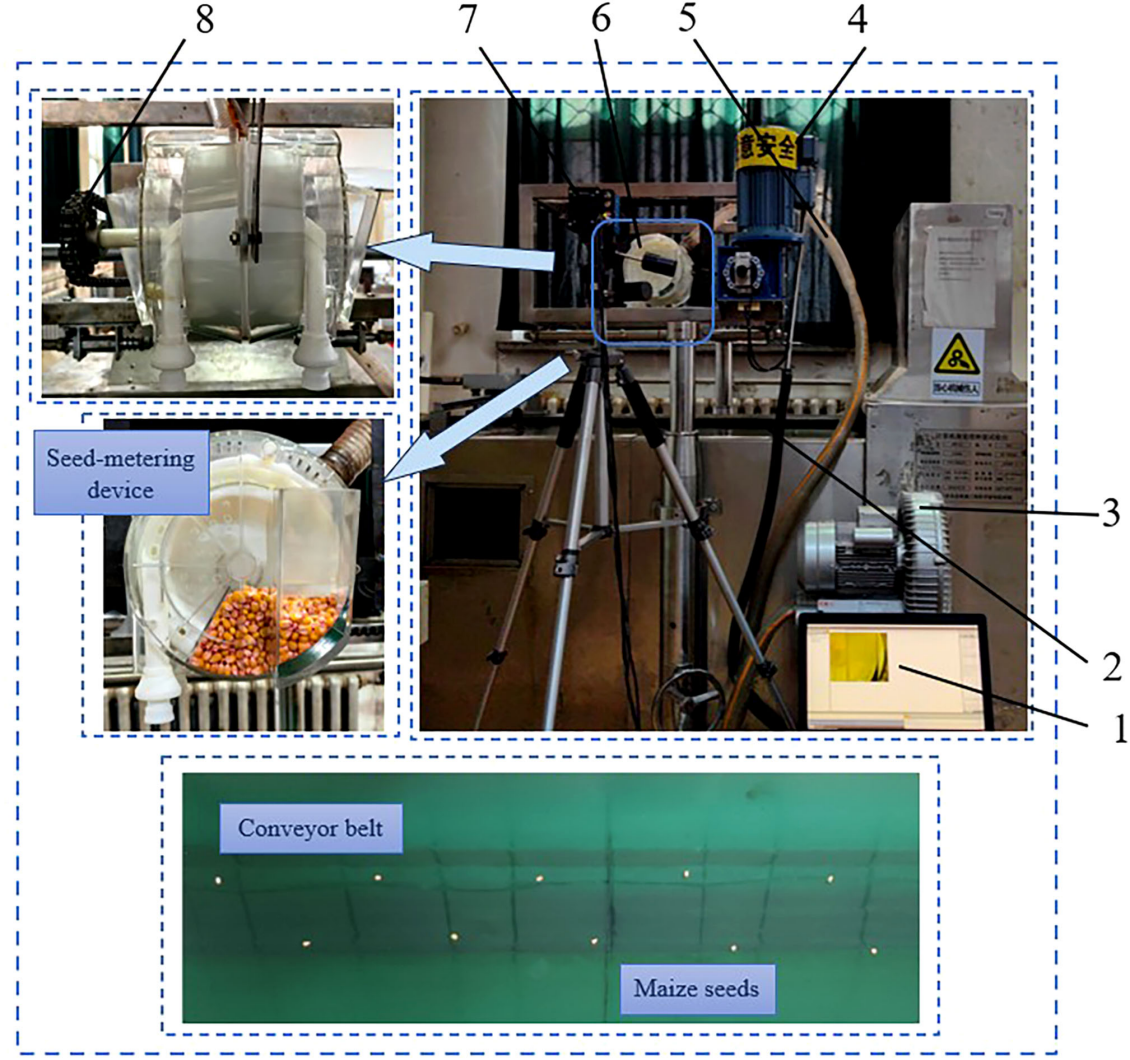
Bench test environment. 1. Data acquisition terminal, 2. Conveyor sprocket, 3. Adjustable pressure fan, 4. Electrical machinery, 5. Pipeline, 6. High-speed precision dual-chamber maize metering device, 7. High-speed camera, 8. Sprocket.

In June 2023, the bench test was conducted in the Engineering Practice Laboratory of Northeast Agricultural University, Harbin, Heilongjiang Province, as shown in **Figure 6**.

The seeding performance test bench was built by Northeast Agricultural University. The metering device was installed on the test bench frame, with the motor serving as the power source. The seed discs were driven by sprockets, and the pipeline connected the metering device to an adjustable pressure blower. Grid paper with each grid measuring 1cm in length was fixed beneath the seed metering device. A high-speed camera (PCO company, Bayern, Germany) was positioned directly above the grid paper to capture the trajectory of maize seeds during the sowing process. The capture field of the high-speed camera was set at 512 mm × 512 mm, with a frame rate of 1000 frames/s and an exposure duration of 990 μs. A tripod was used to stabilize the high-speed camera. The “Demeiya No. 1” maize seeds, widely planted in Northeast China, were selected as the test variety.

#### 2.5.2 Single-factor test

The performance of pneumatic metering devices is mainly affected by operating speed and negative pressure. To explore the effect of these factors on indicators of performance, the study selects negative pressure and operating speed as test factors.

The study analyzed changes in seeding performance under various conditions of operating speed (ranging from 12 to 18 km/h) and negative pressure (ranging from 10 to 16 kPa) based on the requirements of actual seeding and the parameters of the metering device. Single-factor tests were conducted for operating speed and negative pressure to determine the influence of each test factor on seed metering quality. For the operating speed test, the negative pressure was fixed at 13 kPa, and the operating speed was set at seven levels ranging from 12 to 18 km/h. For the negative pressure test, the operating speed was fixed at 15 km/h (seed disc rotation speeds of 33.3 r/min), and the negative pressure was set at seven levels ranging from 10 to 16 kPa.

ISO 7256/1 defines multiples seeding as a distance smaller than half the nominal distance and missed seeding distance a distance longer than one and a half nominal distances. For dense planting with large ridges, the theoretical spacing is 300 mm. Data is collected by using a tape measure to measure the spacing between plants. The evaluation criteria include multiples rate, leakage rate, and qualified rate. Each test group is run five times and the average is taken as the test result.

Based on the results of a preliminary single-factor test, a high-speed camera test was conducted at 14 kPa to investigate the effect of different operating speeds on the sowing process. The trajectory of maize seed drop was analyzed by collecting images of the seed’s descent. The test was designed to select the minimum, intermediate, and maximum values within the reasonable operating speed range as the three levels for the experiment, with operating speeds set at 12, 15, and 18 km/h. This approach enabled a more intuitive examination of the impact of operating speed on the trajectory of the falling maize and facilitated the analysis of the causes of seeding point deviation.

#### 2.5.3 Multi-factor tests

In order to further analyze the seed metering quality and to explore the optimum operational parameters, A two-factor, five-level, orthogonal rotation test was conducted (**Table 1**). Negative pressure and operating speed were used as test factors. The qualified rate and the coefficient of variation were used as test indicators.

**Table 1:** Coding table for test factors.

Code	Test factors	
	Operating speed *X*_1_/(km/h)	Negative pressure *X*_2_/kPa
-1.414	12.172	10.172
-1	13	11
0	15	13
1	17	15
1.414	17.828	15.828

**Table 2:** Parameters of single-chamber metering device and dual-chamber metering device.

Items	Pneumatic high-speed precision metering device	High-speed precision dual-chamber metering device
Number of seeding rows	1	2
Operating speed (km/h)	10	15
Negative pressure (kPa)	4.5	13
Number of holes	26	25
Diameter of seed disc (mm)	166	180
Diameter of holes (mm)	4.4	5
Inclination of seed disc (°)	15	0

Perform Response Surface Analysis on the test results to explore the optimal combination of parameters and comprehensively evaluate the seed metering quality.

#### 2.5.4 Analysis of energy consumption

To analyze the energy consumption of the high-speed precision dual-chamber maize metering device, we compared it with the popular single-chamber pneumatic high-speed precision metering device available in the market. Both types of devices were operated at their optimal working speeds for dense planting operations. Parameters such as drive torque and fan power during the sowing process were measured (**Table 2**). Finally, the energy consumption for operations on one hectare of cultivated land was compared and analyzed.

#### 2.5.5 Data statistics and processing

The calculation formulas for each test indicator are as follows.


(7) $$\left\{ \begin{array}{l} A=\frac{n_{1}}{N^{\prime }}\times 100\%\\ D=\frac{n_{2}}{N^{\prime }}\times 100\%\\ M=\frac{n_{0}}{N^{\prime }}\times 100\%\\ C=\sqrt{\frac{\sum {\left(x-\bar{x}\right)}^{2}}{(N^{'}-1){\bar{x}}^{2}}}\times 100\% \end{array}\right.$$

Where *A* is qualified rate, *D* is multiples rate, *M* is leakage rate, *C* is coefficient variation, *n*_1_ is the quantity of qualified seeded seeds, *n*_2_ is the quantity of seeds for multiple sowing, *n*_0_ is the quantity of seeds missed during sowing, *N*’ is the total quantity of sample intervals, *x* is the seed spacing during the test, *x* is the average value of the sample seed distance.

The relationship between seed disc rotational speed and operating speed is:


(8) $$Z=\frac{60V}{NL}$$

Where, *N* is seed disc rpm, *L* is grain spacing, Z is the quantity of holes, *V* is operating speed.

The formula for calculating energy consumption per hectare during dense planting operations using the metering device is as follows:


(9) $$E=(\frac{NT_{r}}{9550}+P_{d})\frac{80}{VB}$$

Where *E* is total energy consumption, *T_r_* is torque, *B* is the number of seeding rows, and *P_d_* is the power of the fan.

## 3 Results and discussion

### 3.1 Analysis of single-factor test

**Figure 7:**
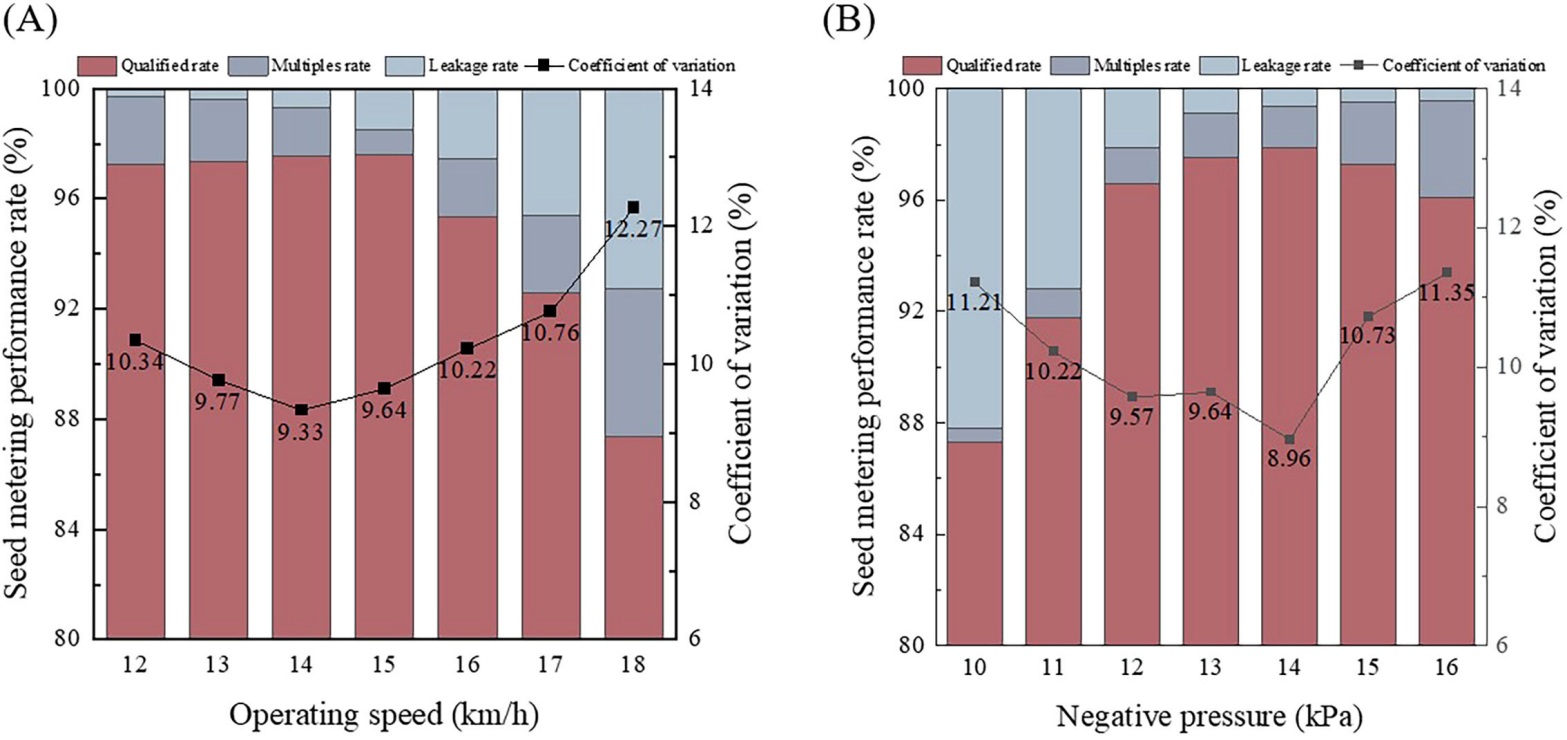
Single-factor test results. **(A)** Effect of the operating speed on the seed metering quality; **(B)** Effect of the Negative Pressure on the seed metering quality.

The impact of operating speed on the qualified rate, multiples rate, leakage rate, and coefficient of variation was analyzed of the single-factor test data (**Figure 7A**).

To determine the relationship between different operating speeds and the leakage rate, multiples rate, qualified rate, and coefficient of variation, a statistic analysis of the test data was performed. With the negative pressure fixed to 13 kPa, the operating speed was increased gradually and the observations recorded. As the operating speed increased, the qualified rate initially showed a slow increase and then a decrease, while the leakage rate gradually increased. The multiples rate initially decreased and then gradually increased, and the coefficient of variation showed a rising tendency.

When the operating speed ranged from 12 to 16 km/h, The qualified rate remained at more than 95.36%, with the highest leakage rate being 2.55%, and the multiples rate not exceeding 2.47%. At 12 km/h, the leakage rate was the lowest, at 0.29%. At an operating speed of 14 km/h, the coefficient of variation was the lowest, at 9.33%. At 15 km/h, the qualified rate was highest, at 97.61%, and the multiples rate was lowest, at 0.88%. At 18 km/h, the qualified rate was at its lowest, at 87.38%, while the leakage rate reached its highest, at 7.27%, and the coefficient of variation also peaked, at 12.27%.

Analyzing the test results, at 12 km/h operating speed, the test results showed an excellent qualified rate, but the multiples rate was also higher. At lower operating speeds, the seeding discs rotate more slowly, resulting in an excessively long time for the holes in the seed filling zone to fill. In addition, due to the slower rpm of the seed discs, the excess adsorbed seed is not sufficiently cleared by the seed clearing wheels, resulting in a higher multiples rate. At 18 km/h operating speed, the seed metering performance was poor, with a significant increase in multiples and miss rates. This was due to the high operating speed, which causes excessive collision and compression between the seed and the seed clearing wheels during the clearing process, leading to excessive seed clearing. Furthermore, at the entrance of the seed discharge tube, seeds may collide, causing delayed seed discharge and multiple seeds falling simultaneously, resulting in an increased multiples rate.

The effect of negative pressure on each indicator was analyzed through statistics analysis of the single factor test data (**Figure 7B**).

To determine the relationship between different negative pressure and the qualified rate, leakage rate, multiple rate and coefficient of variation, a statistic analysis of the test data was performed. With the operating speed fixed to 15 km/h, the negative pressure was gradually increased while recording corresponding test data. The quality of the seed filling improved with increasing negative pressure. The qualified rate initially increased rapidly, followed by a gradual decline. The multiples rate increased gradually, while the leakage rate decreased gradually. The coefficient of variation initially decreased before increasing.

When the negative pressure ranged from 10 to 16 kPa, the qualified rate continued to be above 87.26%. The leakage rate did not exceed 12.17% and the multiples rate did not exceed 3.33%. At 14 kPa, the qualified rate was the highest at 97.91%, with the lowest coefficient of variation at 8.96%. As the negative pressure increased, there was a slight decrease in the qualified rate. The leakage rate gradually decreased, reaching its minimum value of 0.43% at a pressure of 16 kPa. Meanwhile, the coefficient of variation and the multiples rate peaked, reaching 11.35% and 3.48% respectively. All performance indicators could be maintained at a relatively optimal level within the negative pressure range of 13 to 16 kPa. The operational efficiency was optimal at 14 kPa but gradually declined thereafter.

When the test data was analyzed, it was observed that the leakage rate was relatively high at 10 kPa. This is due to the weaker suction ability at this pressure, leading to low seed filling capability. Seeds in the seed filling zone exhibit unstable adhesion, leading to detachment during collision with the seed clearing wheels. Excessive negative pressure can cause multiple seeds to be adsorbed within a single hole, and excess seeds may not be effectively cleared by the seed clearing wheels. Furthermore, seeds at the end of the seed sowing zone may have difficulty detaching, resulting in collisions between two or more seeds at the entrance of the seed discharge tube. Therefore, the multiples rate is excessively high, which significantly reduces the seeding performance.

The test data shows that when the negative pressure is set to 13 kPa and the operating speed is between 12 - 16 km/h, the seeding performance is excellent, with a qualified rate above 95%. Similarly, at an operating speed of 15 km/h and with a negative pressure ranging from 12 to 16 kPa, the seeding performance remains excellent.

### 3.2 Analysis of seed falling test

The dense planting pattern places high demands on the seed drop locations. As the operating speed of the planter increases, the maize falls at an accelerated rate, reducing the falling time. Simultaneously, the direction of seed sowing and the trajectory of seeds also change, affecting the sowing position, ultimately influencing the seeding effect of the dense planting pattern. Therefore, the process of sowing needs to be studied.

**Figure 8:**
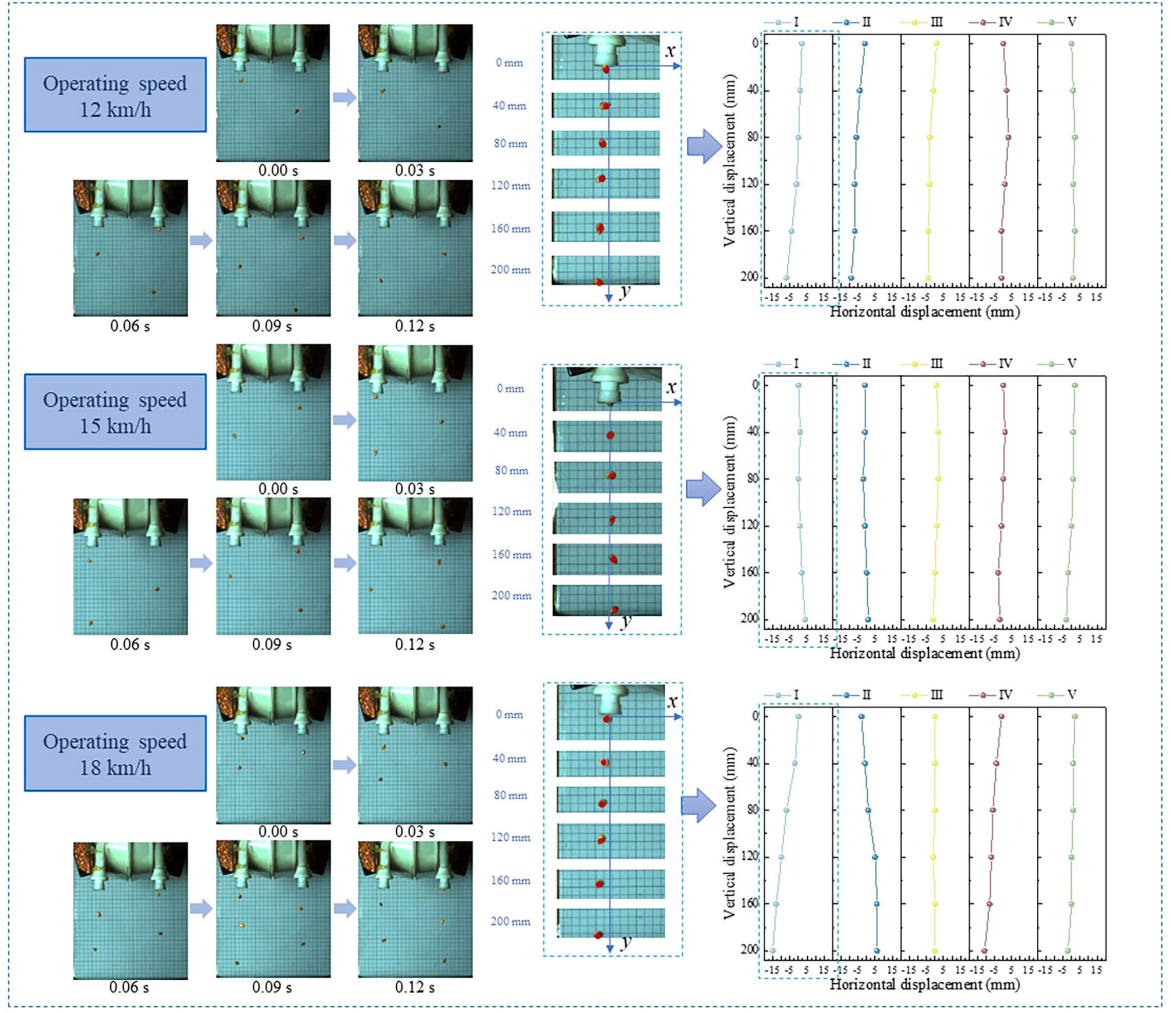
Trajectory and displacement of seeds at different operating speeds.

The trajectory of the maize seed was recorded at different operating speeds and the data was statistically analyzed (**Figure 8**).

The trajectory of maize seeds falling at different operating speeds was observed by processing the images captured by high-speed cameras. The intra-row displacement of maize seeds was assessed during the sowing process by marking each frame of seeds to determine their motion under operating speed. Analysis of the displacement images revealed that at operating speeds of 12, 15, and 18 km/h, the average ± standard deviation of maize seed falling displacement were -1.53 ± 3.22 mm, 0.10 ± 1.58 mm, and -2.30 ± 5.15 mm, respectively. Test data indicated that at 15 km/h operating speed, the average and variance of maize seed falling displacement were the smallest, suggesting the most reasonable and stable trajectory for maize seed descent.

Analyzing the images captured during the high-speed camera test (**Figure 8**), At 12 km/h, maize seeds exhibit difficulty in falling along an approximately straight sowing trajectory, resulting in a considerable amount of falling offset. The primary reason is that when the operating speed is low, the seed discs rotation speed is also low. This results in the maize being ejected from the end of the seed guide tubes at a lower speed. During the descent process, the center of gravity shifts, leading to rotation, ultimately resulting in an unstable sowing trajectory. The mean and standard deviation of the seed drop displacements were observed to be the highest at an operating speed of 18 km/h. The seeds were observed to have sufficient initial velocity to reduce the effects of rotation during the seed drop. The initial velocity of the maize seeds is sufficient to minimize the effect of rotation during descent. However, the high operating speed results in an increase in the rotational speed of the seed disc, which may prevent some seeds from disengaging from the disc in time to collide with the inlet of the seed metering tube at the initial stage of the sowing process. This collision imparts a horizontal velocity to the seeds, resulting in an excessive average drop displacement of the maize seeds. Furthermore, the standard deviation of the maize seed fall displacement increases due to the uncertainty of the direction after the collision.

### 3.3 Analysis of optimum operating parameters

A two-factor, five-level orthogonal rotation combination test was performed using operating speed and negative pressure as test factors, with qualified rate and coefficient of variation as test indicators, followed by response surface analysis (**Table 3**).

**Table 3:** Test data.

No.	Factors		Indicators	
	Operating speed *X*_1_/(km/h)	Negative pressure *X*_2_/kPa	Qualified rate *Y*_1_/%	Coefficient of variation *Y*_2_/%
1	-1 (13)	-1 (11)	92.56	14.67
2	1 (17)	-1	84.32	19.85
3	-1	1 (15)	95.28	11.94
4	1	1	93.11	13.78
5	-1.414 (12.1716)	0 (13)	97.27	10.55
6	1.414 (17.8284)	0	88.89	15.86
7	0 (15)	-1.414 (10.1716)	86.47	19.24
8	0	1.414 (15.8284)	94.36	13.42
9	0	0	97.64	9.42
10	0	0	97.66	9.18
11	0	0	97.82	8.62
12	0	0	97.53	9.17
13	0	0	97.31	9.73

The test results listed as **Table 4**. The regression model was highly significant and the lack of fit term was non-significant, suggesting that the model is accurate and effective.

**Table 4:** Analysis of variance results.

Source		Sum of squares	Degrees of freedom	Mean square	F-value	P-value	Significance
*Y*_1_	Model	255.47	5	51.09	454.36	< 0.0001	**
	*X*_1_	61.94	1	61.94	550.86	< 0.0001	**
	*X*_2_	64.23	1	64.23	571.19	< 0.0001	**
	*X*_1_ *X*_2_	9.21	1	9.21	81.91	< 0.0001	**
	*X*_1_^2^	38.86	1	38.86	345.57	< 0.0001	**
	*X*_2_^2^	95.03	1	95.03	854.07	< 0.0001	**
	Residual	0.7872	7	0.1125			
	Lack of fit	0.6449	3	0.2150	6.04	0.0574	#
	Pure error	0.1423	4	0.0356			
	Cor total	256.25	12				
*Y*_2_	Model	174.81	5	34.96	270.22	< 0.0001	**
	*X*_1_	26.39	1	26.39	203.95	< 0.0001	**
	*X*_2_	36.26	1	36.26	280.22	< 0.0001	**
	*X*_1_ *X*_2_	2.79	1	2.79	21.56	0.0024	**
	*X*_1_^2^	29.62	1	29.62	228.97	< 0.0001	**
	*X*_2_^2^	91.47	1	91.47	706.96	< 0.0001	**
	Residual	0.9057	7	0.1294			
	Lack of fit	0.2416	3	0.0805	0.4850	0.7107	#
	Pure error	0.6641	4	0.1660			
	Cor total	175.71	12				

** represents extremely significant (p< 0.01), * represents significant (p< 0.05), and # represents insignificant (p > 0.05).

**Figure 9:**
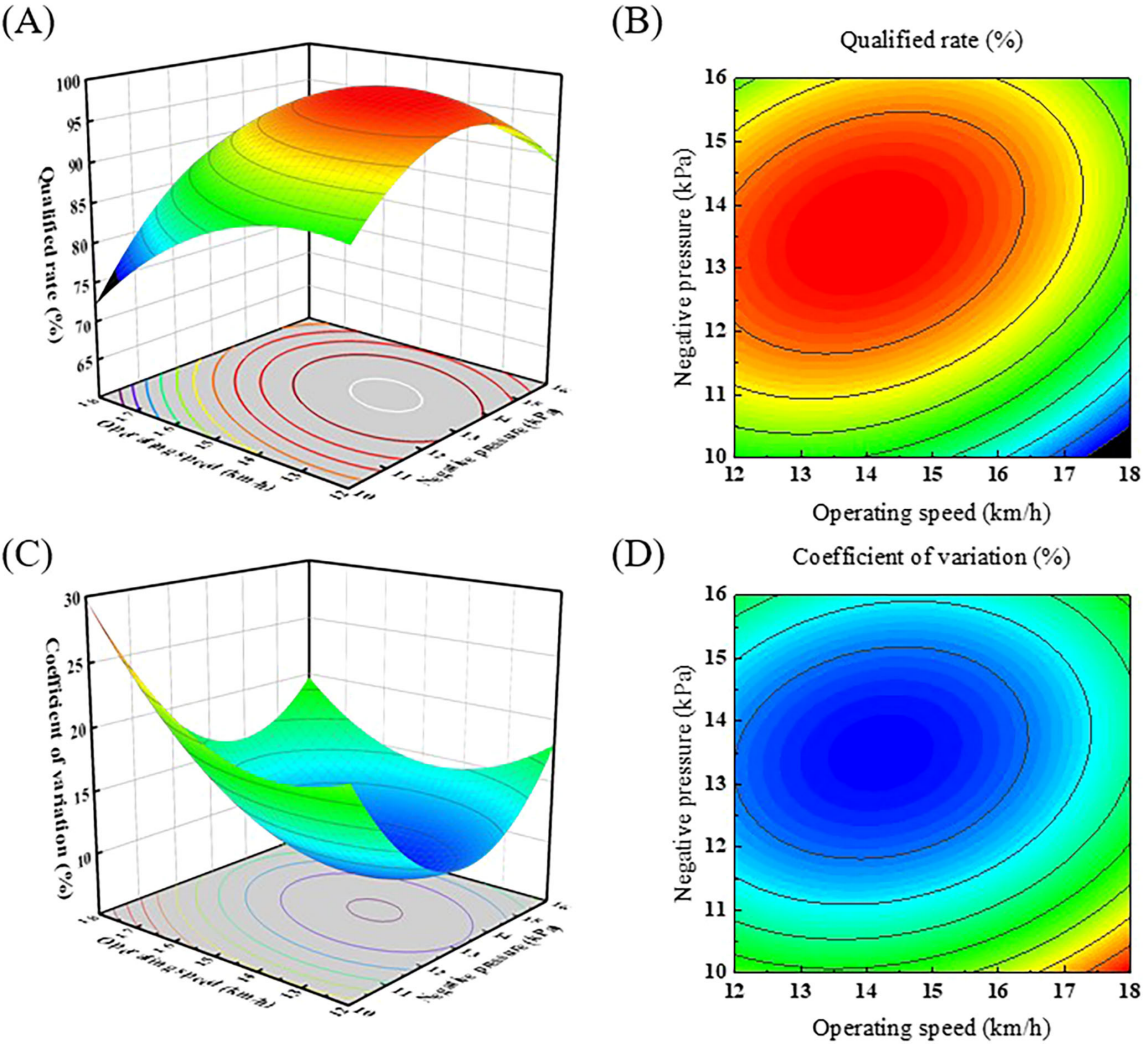
Response surface graph and contour graph of qualified rate and coefficient of variation. **(A)** Response surface graph of qualified rate; **(B)** Contour graph of qualified rate; **(C)** Response surface graph of coefficient of variation; **(D)** Contour graph of coefficient of variation.


Based on the F-value, the qualified rate is affected by the following factors in this sequence: *X*_2_ > *X*_1_ > *X*_1_ *X*_2_. The coefficient of variation is affected by the following factors in this sequence: *X*_2_ > *X*_1_ > *X*_1_ *X*_2_. Response surface plots were generated to optimize factor settings and understand the interactions and effects among them (**Figure 9**).

(10) $$\{\begin{array}{c} Y_{1}=-115.0808+11.4031X_{1}+19.7501X_{2}+0.3794X_{1}X_{2}-0.5909X_{1}^{2}-0.9240X_{2}^{2}\\ Y_{2}=238.0165-11.8554X_{1}-21.5030X_{2}-0.2088X_{1}X_{2}+0.5159X_{1}^{2}+0.9065X_{2}^{2} \end{array}$$

The interaction of negative pressure and operating speed on the qualified rate is shown in **Figure 9**. In the *X*_1_ *X*_2_ interaction surface, the qualified rate changes with an initial slow rise and then a rapid fall as the operating speed increases. At relatively low operating speeds, an increase in negative pressure leads to an initial increase followed by a reduction of the qualified rate. Conversely, when the operating speed is relatively high, the qualified rate shows an initially increasing trend, which then tends to level off as the operating speed increases. This suggests that the interaction between negative pressure and operating speed has a significant effect on the qualified rate.

The interaction of operating speed and negative pressure on the coefficient of variation is shown in **Figure 9**. In the *X*_1_ *X*_2_ interaction surface, the coefficient of variation shows a tendency to decrease initially and then to increase with increasing operating speed. For different negative pressure levels, the magnitude of change in the coefficient of variation varies as the operating speed increases. This suggests that the interaction of negative pressure and operating speed also has a significant effect on the coefficient of variation.

To achieve the optimum combinations, the objective is to maximize the qualified rate and minimize the coefficient of variation within the parameter design range. Equation 11 is used as the objective function.


(11) $$\{\begin{array}{l} \max Y_{1}(X_{1},X_{2})\\ \min Y_{2}(X_{1},X_{2})\\ s.t.\{\begin{array}{c} 12.0\text{km/h }<\;X_{1}\;<18.0\text{km/h}\\ 10.0\text{kPa }<\;X_{2}\;<16.0\text{kPa } \end{array} \end{array}$$

According to the regression equation model, the predicted optimal conditions were 14.219 km/h operating speed and 13.497 kPa negative pressure. These values were corrected to 14.2 km/h operating speed and 13.5 kPa negative pressure to reflect actual conditions. The combination resulted in a qualified rate of 98.65%, and a coefficient variation of 8.61%.

Three parallel tests were conducted using the optimal parameter combination derived from theoretical optimization. The obtained qualified rate was 97.93%, and the coefficient variation was 8.97%. The results were within a 5% deviation from the predicted values, confirming a good correlation between predicted and test values.

### 3.4 Analysis of energy consumption

The test results of the single-chamber metering device and the high-speed precision dual-chamber maize metering device at their optimal operating speeds are shown in **Table 5**.

**Table 5:** Test results.

Items	Dual-chamber maize metering device	Single-chamber metering device
Operating speed (km/h)	15	10
Operation time (h)	0.74	2.22
Rotation speed of seed disc(r/min)	33.33	21.37
Torsion (N m)	1.52	1.38
Fan rated power (W)	370	180
Seed disc rotating power (W)	5.3	3.1
Total power (W)	375.3	183.1
Energy consumption per hectare (kJ)	997.8	1463.3

**Figure 10:**
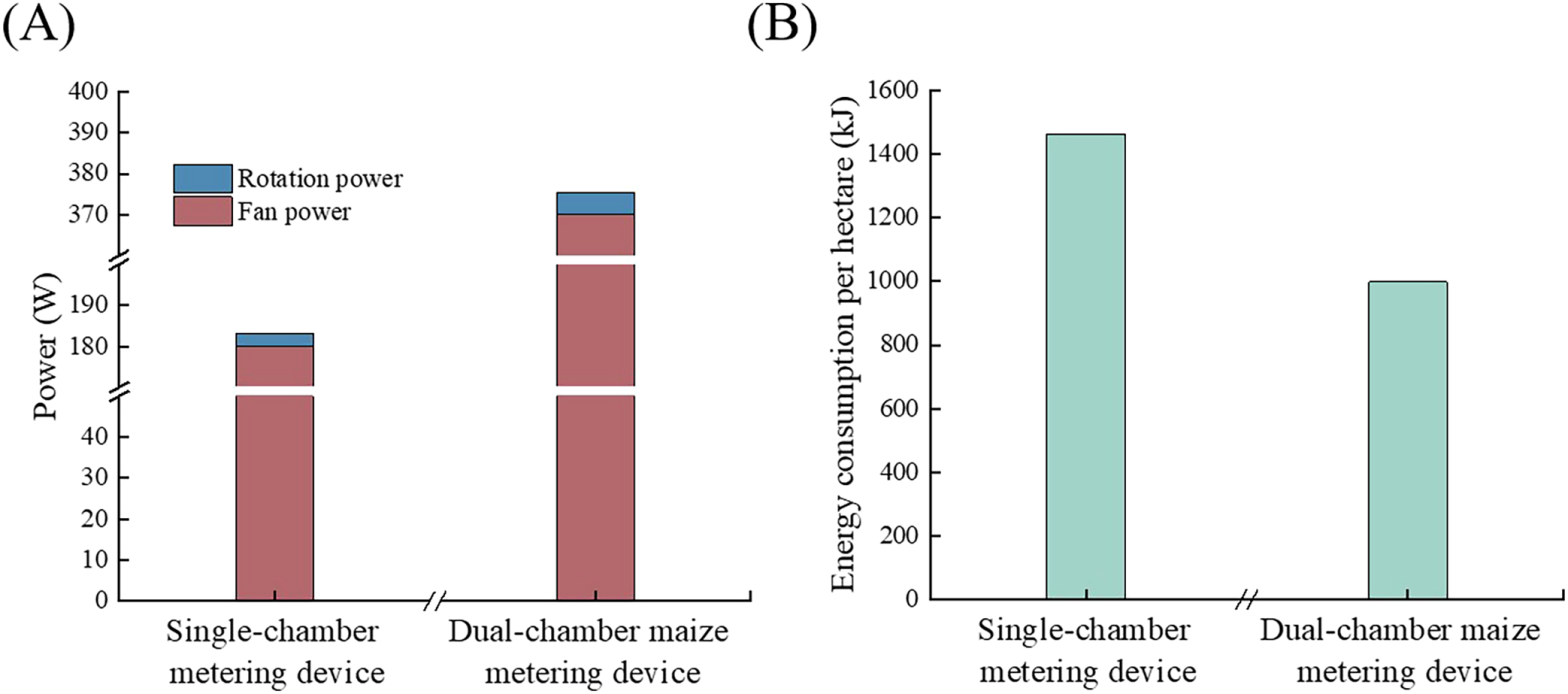
Energy consumption and power requirement for dual and single chamber maize metering devices. **(A)** Comparison of power; **(B)** Comparison of energy consumption.

The test results show that the power consumption of the vacuum fan accounts for the majority of the total power consumption (**Figure 10**). The power of the dual chamber maize metering device is higher compared to the power of the single chamber seed metering device. The main reason for this is that the dual-chamber metering device employs a single air duct to supply vacuum to both chambers., requiring a larger negative pressure compared to the single-chamber metering device, thus necessitating a larger power capacity for the negative pressure fan.

Comparing the energy consumption of the single-chamber metering device and the dual-chamber maize metering device for dense planting on one hectare, the energy consumption of the dual-chamber seed metering device for the same area of operation is reduced by approximately 32% compared to the single-chamber metering device. The primary reason is that the dual-chamber maize metering device can complete seeding for two rows at once, while operating at a faster speed. With a shorter operating time for the same area of operation, the total energy consumption is significantly reduced.

### 3.5 Discussion

In comparison with the equal spacing planting pattern for maize, the dense planting pattern with standard ridges maximizes the utilization of land, water, and fertilizer resources by reducing row spacing and increasing plant density, ensuring clean production while achieving stable and increased grain yields. This planting pattern has been widely used in northeastern China, but there has been limited research on planting machines specifically designed for this pattern.

Compared to single-chamber metering devices, the high-speed precision dual-chamber maize metering device adopts the dual discs method installed in opposite and staggered positions. The advantage lies in the simultaneous seeding of two rows on a single ridge by the dual plates, achieving high operating speed with lower seed disc rotation speed, ensuring operational effectiveness, and enhancing operational efficiency (Tang et al., 2024; Zhao et al., 2024).

In the research field of seed metering devices, the CFD-DEM coupling method is widely used. Han et al. (2018) proposed an internally filled pneumatic maize metering device. Application of the coupled DEM - CFD method, the movement of seeds in an airflow field was simulated, optimizing the structural design features and improving its operational performance.

The maize high-speed precision dual-chamber maize metering device proposed in this study necessitates the application of elevated negative pressure, due to the single air channel that is required to provide negative pressure for the dual side chambers, resulting in more energy consumption.

During the test, it was found that the limitations of the 3D printing process resulted in insufficient airtightness of the metering device during operation, requiring higher negative pressure. In the next phase of the research, the method of making the metering device will be modified. Add rubber seals between the airflow channel and the seed discs to enhance airtightness, reduce the need for negative pressure, and further reduce the level of energy consumption of the high-speed precision dual-chamber maize metering device during the production process of the dense planting pattern with standard ridges.

During field operations, it is necessary to consider the effect of machine vibration on seeding performance. Vibration can dislodge seed adhering to the seed disc, and leading to collisions between the seed and the sowing tube. This ultimately affects seeding performance, leading to insufficient seeding to meet the agronomic requirements of the dense planting pattern of standard ridges. The next step in the research will consist of field testing the seeder under different combinations of operating parameters. Sensors will be installed on the frame and planter to collect vibration data. In conjunction with CFD-DEM simulation experiments, the effect of vibration on seeding performance will be analyzed and the vibration data will be fully analyzed with respect to the forces and motions of the seed in order to improve the design of the device.

## 4 Conclusion

1. At 13 kPa negative pressure and operating speeds ranging from 12 to 16 km/h, the qualified rate is consistently above 95.36%, meeting the demands of high-speed seeding.
2. Compared to conventional single-chamber metering device, the energy consumption of the high-speed precision dual-chamber maize metering device is approximately 32% lower for dense planting patterns with standard ridges in the same area.
3. The optimum operational conditions are 14.2 km/h operating speed and 13.5 kPa negative pressure. Verification of the optimum conditions yielded a qualified rate of 97.93% and a coefficient variation of 8.97%.
4. This study innovatively proposes a high-speed precision dual-chamber maize metering device tailored for the dense planting pattern with standard ridges, reducing energy consumption in maize production. It provides new insights for promoting the sustainable development of agricultural production and offers a reference for research into clean production methods for different agronomic patterns.

## Data Availability

The original contributions presented in the study are included in the article/supplementary material. Further inquiries can be directed to the corresponding authors.
